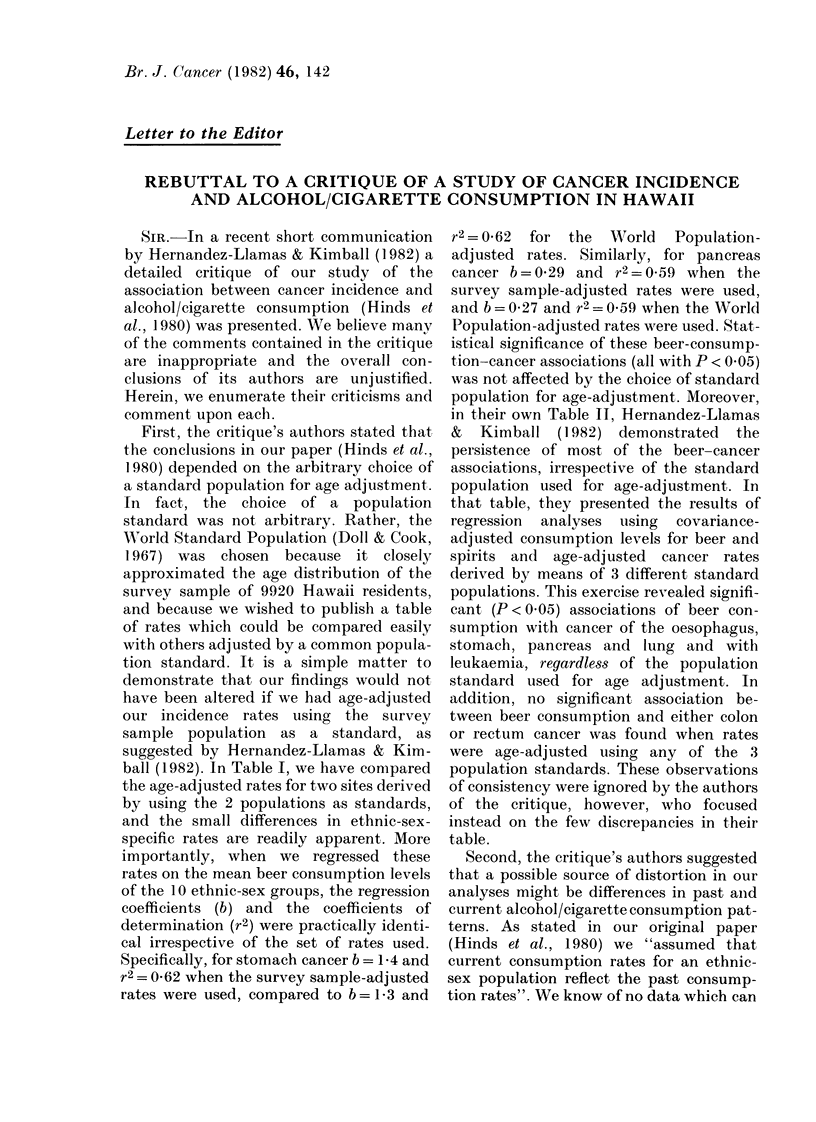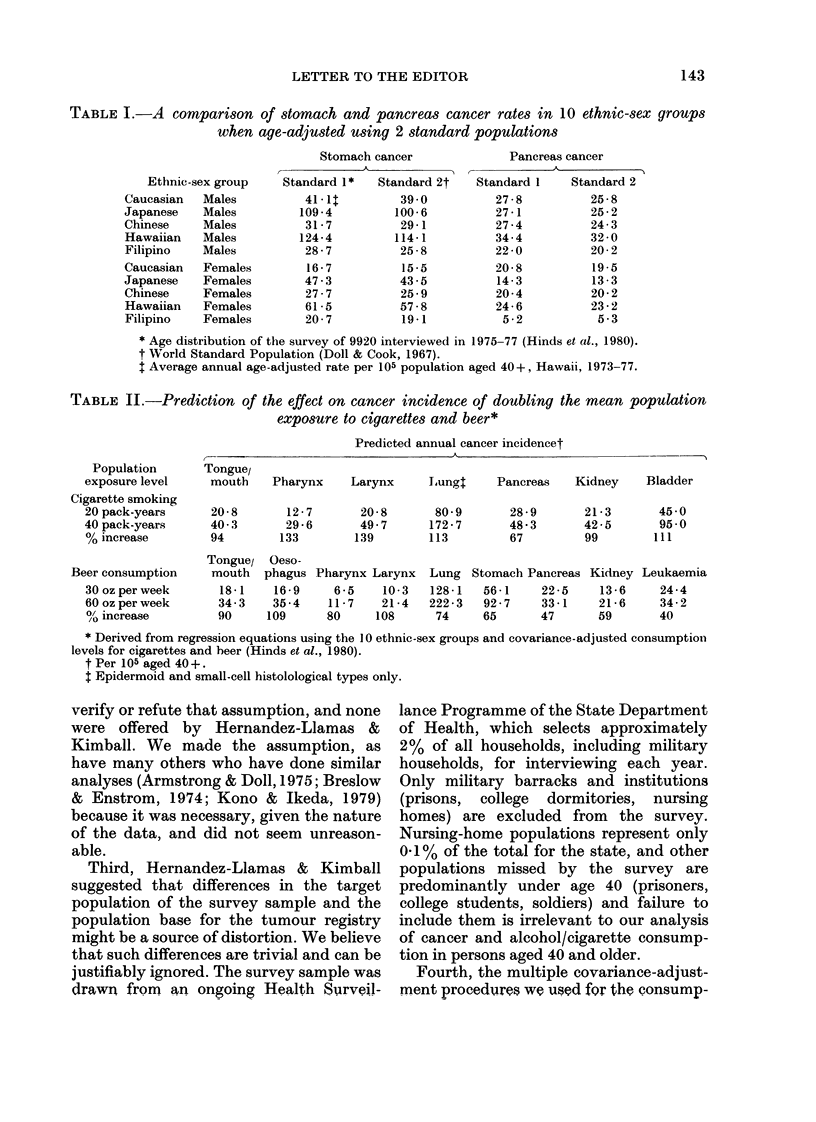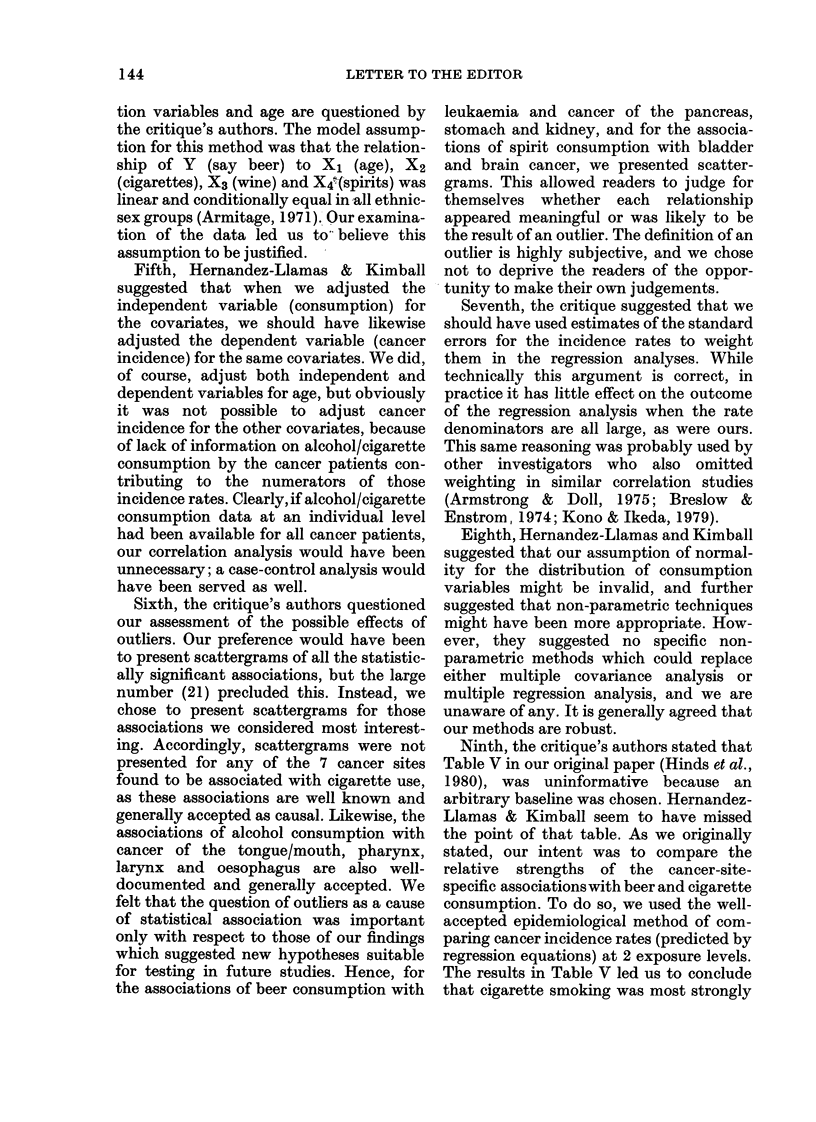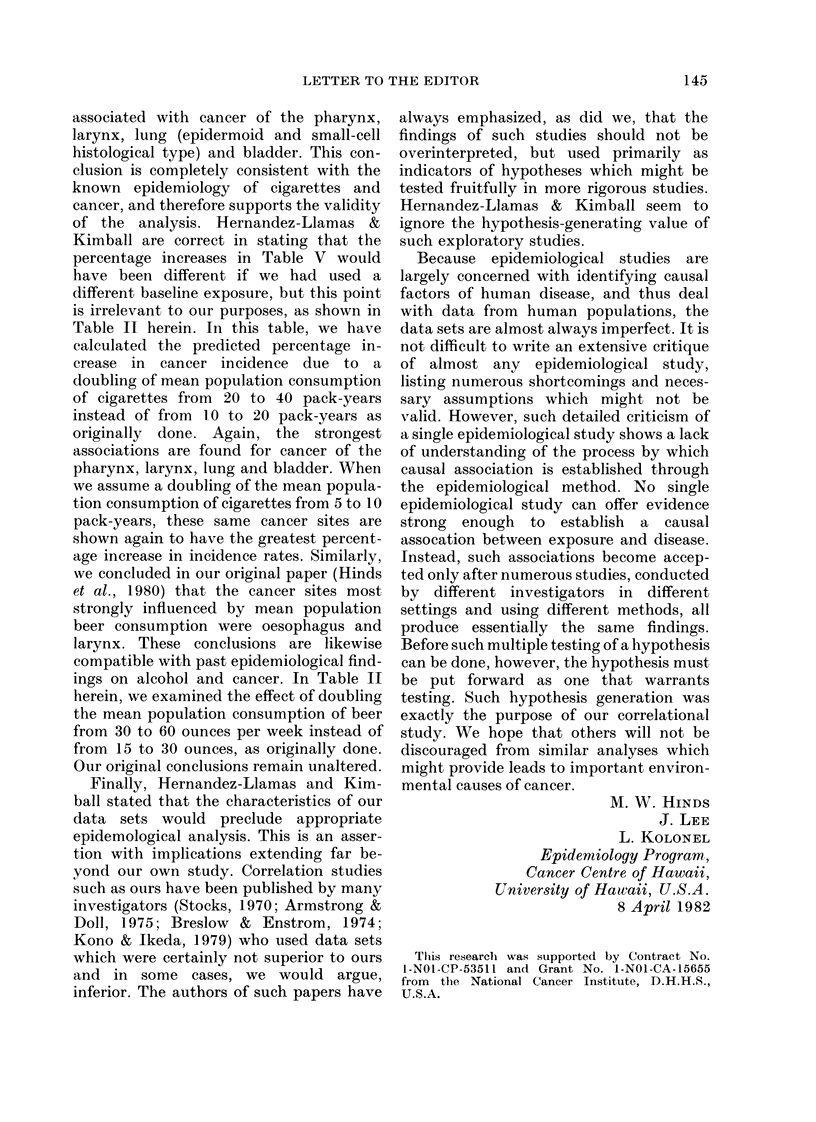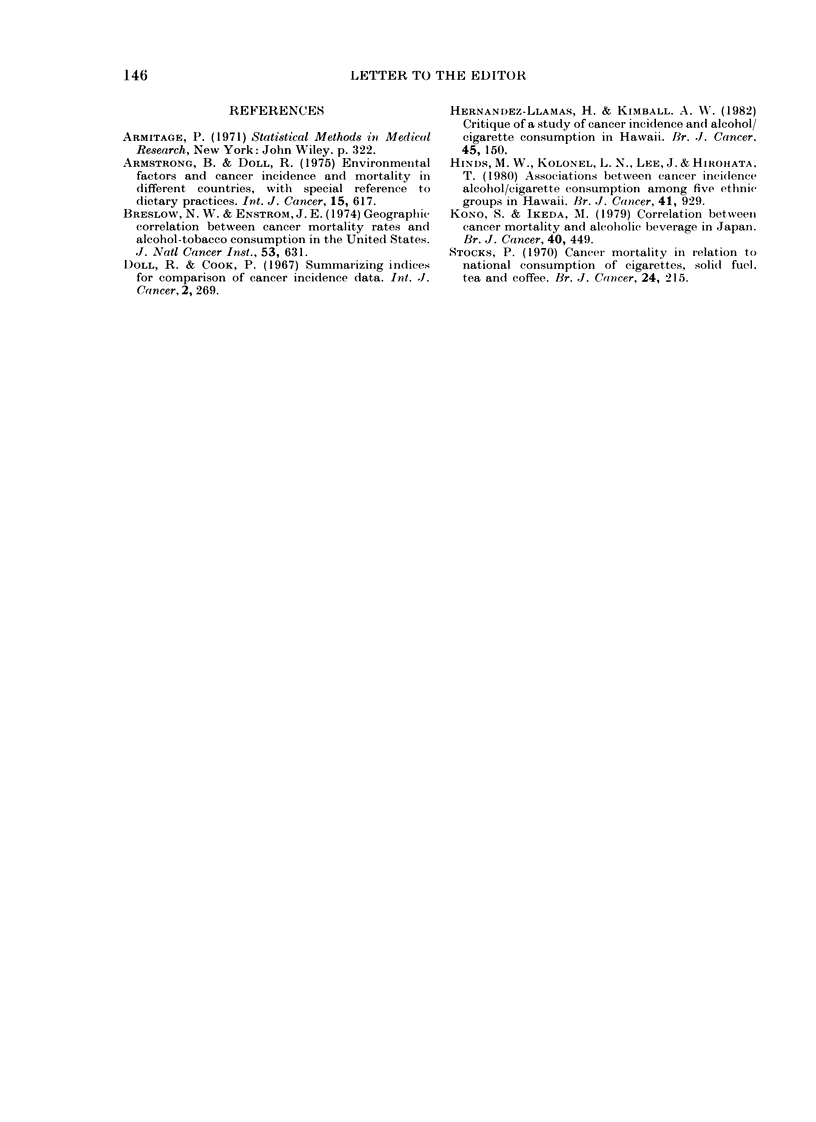# Rebuttal to a critique of a study of cancer incidence and alcohol/cigarette consumption in Hawaii.

**DOI:** 10.1038/bjc.1982.176

**Published:** 1982-07

**Authors:** M. W. Hinds, J. Lee, L. Kolonel


Br. J. Cancer (1982) 46, 142

*Letter to the Editor*

### REBUTTAL TO A CRITIQUE OF A STUDY OF CANCER INCIDENCE AND ALCOHOL/CIGARETTE CONSUMPTION IN HAWAII

SIR.—In a recent short communication by Hernandez-Llamas & Kimball (1982) a detailed critique of our study of the association between cancer incidence and alcohol/cigarette consumption (Hinds *et al.*, 1980) was presented. We believe many of the comments contained in the critique are inappropriate and the overall conclusions of its authors are unjustified. Herein, we enumerate their criticisms and comment upon each.

First, the critique's authors stated that the conclusions in our paper (Hinds *et al.*, 1980) depended on the arbitrary choice of a standard population for age adjustment. In fact, the choice of a population standard was not arbitrary. Rather, the World Standard Population (Doll & Cook, 1967) was chosen because it closely approximated the age distribution of the survey sample of 9920 Hawaii residents, and because we wished to publish a table of rates which could be compared easily with others adjusted by a common population standard. It is a simple matter to demonstrate that our findings would not have been altered if we had age-adjusted our incidence rates using the survey sample population as a standard, as suggested by Hernandez-Llamas & Kimball (1982). In Table I, we have compared the age-adjusted rates for two sites derived by using the 2 populations as standards, and the small differences in ethnic-sex-specific rates are readily apparent. More importantly, when we regressed these rates on the mean beer consumption levels of the 10 ethnic-sex groups, the regression coefficients ($b$) and the coefficients of determination ($r^2$) were practically identical irrespective of the set of rates used. Specifically, for stomach cancer $b = 1{\cdot}4$ and $r^2 = 0{\cdot}62$ when the survey sample-adjusted rates were used, compared to $b = 1{\cdot}3$ and $r^2 = 0{\cdot}62$ for the World Population-adjusted rates. Similarly, for pancreas cancer $b = 0{\cdot}29$ and $r^2 = 0{\cdot}59$ when the survey sample-adjusted rates were used, and $b = 0{\cdot}27$ and $r^2 = 0{\cdot}59$ when the World Population-adjusted rates were used. Statistical significance of these beer-consumption–cancer associations (all with $P < 0{\cdot}05$) was not affected by the choice of standard population for age-adjustment. Moreover, in their own Table II, Hernandez-Llamas & Kimball (1982) demonstrated the persistence of most of the beer–cancer associations, irrespective of the standard population used for age-adjustment. In that table, they presented the results of regression analyses using covariance-adjusted consumption levels for beer and spirits and age-adjusted cancer rates derived by means of 3 different standard populations. This exercise revealed significant ($P < 0{\cdot}05$) associations of beer consumption with cancer of the oesophagus, stomach, pancreas and lung and with leukaemia, *regardless* of the population standard used for age adjustment. In addition, no significant association between beer consumption and either colon or rectum cancer was found when rates were age-adjusted using any of the 3 population standards. These observations of consistency were ignored by the authors of the critique, however, who focused instead on the few discrepancies in their table.

Second, the critique's authors suggested that a possible source of distortion in our analyses might be differences in past and current alcohol/cigarette consumption patterns. As stated in our original paper (Hinds *et al.*, 1980) we "assumed that current consumption rates for an ethnic-sex population reflect the past consumption rates". We know of no data which can

TABLE I.—*A comparison of stomach and pancreas cancer rates in* 10 *ethnic-sex groups when age-adjusted using* 2 *standard populations*

| Ethnic-sex group | | Stomach cancer Standard 1* | Stomach cancer Standard 2† | Pancreas cancer Standard 1 | Pancreas cancer Standard 2 |
|---|---|---|---|---|---|
| Caucasian | Males | 41·1‡ | 39·0 | 27·8 | 25·8 |
| Japanese | Males | 109·4 | 100·6 | 27·1 | 25·2 |
| Chinese | Males | 31·7 | 29·1 | 27·4 | 24·3 |
| Hawaiian | Males | 124·4 | 114·1 | 34·4 | 32·0 |
| Filipino | Males | 28·7 | 25·8 | 22·0 | 20·2 |
| Caucasian | Females | 16·7 | 15·5 | 20·8 | 19·5 |
| Japanese | Females | 47·3 | 43·5 | 14·3 | 13·3 |
| Chinese | Females | 27·7 | 25·9 | 20·4 | 20·2 |
| Hawaiian | Females | 61·5 | 57·8 | 24·6 | 23·2 |
| Filipino | Females | 20·7 | 19·1 | 5·2 | 5·3 |

* Age distribution of the survey of 9920 interviewed in 1975–77 (Hinds *et al.*, 1980).
† World Standard Population (Doll & Cook, 1967).
‡ Average annual age-adjusted rate per $10^5$ population aged 40+, Hawaii, 1973–77.

TABLE II.—*Prediction of the effect on cancer incidence of doubling the mean population exposure to cigarettes and beer**

Predicted annual cancer incidence†

| Population exposure level | Tongue/mouth | Pharynx | Larynx | Lung‡ | Pancreas | Kidney | Bladder |
|---|---|---|---|---|---|---|---|
| Cigarette smoking | | | | | | | |
| 20 pack-years | 20·8 | 12·7 | 20·8 | 80·9 | 28·9 | 21·3 | 45·0 |
| 40 pack-years | 40·3 | 29·6 | 49·7 | 172·7 | 48·3 | 42·5 | 95·0 |
| % increase | 94 | 133 | 139 | 113 | 67 | 99 | 111 |

| Beer consumption | Tongue/mouth | Oesophagus | Pharynx | Larynx | Lung | Stomach | Pancreas | Kidney | Leukaemia |
|---|---|---|---|---|---|---|---|---|---|
| 30 oz per week | 18·1 | 16·9 | 6·5 | 10·3 | 128·1 | 56·1 | 22·5 | 13·6 | 24·4 |
| 60 oz per week | 34·3 | 35·4 | 11·7 | 21·4 | 222·3 | 92·7 | 33·1 | 21·6 | 34·2 |
| % increase | 90 | 109 | 80 | 108 | 74 | 65 | 47 | 59 | 40 |

* Derived from regression equations using the 10 ethnic-sex groups and covariance-adjusted consumption levels for cigarettes and beer (Hinds *et al.*, 1980).
† Per $10^5$ aged 40+.
‡ Epidermoid and small-cell histolological types only.

verify or refute that assumption, and none were offered by Hernandez-Llamas & Kimball. We made the assumption, as have many others who have done similar analyses (Armstrong & Doll, 1975; Breslow & Enstrom, 1974; Kono & Ikeda, 1979) because it was necessary, given the nature of the data, and did not seem unreasonable.

Third, Hernandez-Llamas & Kimball suggested that differences in the target population of the survey sample and the population base for the tumour registry might be a source of distortion. We believe that such differences are trivial and can be justifiably ignored. The survey sample was drawn from an ongoing Health Surveillance Programme of the State Department of Health, which selects approximately 2% of all households, including military households, for interviewing each year. Only military barracks and institutions (prisons, college dormitories, nursing homes) are excluded from the survey. Nursing-home populations represent only 0·1% of the total for the state, and other populations missed by the survey are predominantly under age 40 (prisoners, college students, soldiers) and failure to include them is irrelevant to our analysis of cancer and alcohol/cigarette consumption in persons aged 40 and older.

Fourth, the multiple covariance-adjustment procedures we used for the consumption variables and age are questioned by the critique's authors. The model assumption for this method was that the relationship of Y (say beer) to $X_1$ (age), $X_2$ (cigarettes), $X_3$ (wine) and $X_4$ (spirits) was linear and conditionally equal in all ethnic-sex groups (Armitage, 1971). Our examination of the data led us to believe this assumption to be justified.

Fifth, Hernandez-Llamas & Kimball suggested that when we adjusted the independent variable (consumption) for the covariates, we should have likewise adjusted the dependent variable (cancer incidence) for the same covariates. We did, of course, adjust both independent and dependent variables for age, but obviously it was not possible to adjust cancer incidence for the other covariates, because of lack of information on alcohol/cigarette consumption by the cancer patients contributing to the numerators of those incidence rates. Clearly, if alcohol/cigarette consumption data at an individual level had been available for all cancer patients, our correlation analysis would have been unnecessary; a case-control analysis would have been served as well.

Sixth, the critique's authors questioned our assessment of the possible effects of outliers. Our preference would have been to present scattergrams of all the statistically significant associations, but the large number (21) precluded this. Instead, we chose to present scattergrams for those associations we considered most interesting. Accordingly, scattergrams were not presented for any of the 7 cancer sites found to be associated with cigarette use, as these associations are well known and generally accepted as causal. Likewise, the associations of alcohol consumption with cancer of the tongue/mouth, pharynx, larynx and oesophagus are also well-documented and generally accepted. We felt that the question of outliers as a cause of statistical association was important only with respect to those of our findings which suggested new hypotheses suitable for testing in future studies. Hence, for the associations of beer consumption with leukaemia and cancer of the pancreas, stomach and kidney, and for the associations of spirit consumption with bladder and brain cancer, we presented scattergrams. This allowed readers to judge for themselves whether each relationship appeared meaningful or was likely to be the result of an outlier. The definition of an outlier is highly subjective, and we chose not to deprive the readers of the opportunity to make their own judgements.

Seventh, the critique suggested that we should have used estimates of the standard errors for the incidence rates to weight them in the regression analyses. While technically this argument is correct, in practice it has little effect on the outcome of the regression analysis when the rate denominators are all large, as were ours. This same reasoning was probably used by other investigators who also omitted weighting in similar correlation studies (Armstrong & Doll, 1975; Breslow & Enstrom, 1974; Kono & Ikeda, 1979).

Eighth, Hernandez-Llamas and Kimball suggested that our assumption of normality for the distribution of consumption variables might be invalid, and further suggested that non-parametric techniques might have been more appropriate. However, they suggested no specific non-parametric methods which could replace either multiple covariance analysis or multiple regression analysis, and we are unaware of any. It is generally agreed that our methods are robust.

Ninth, the critique's authors stated that Table V in our original paper (Hinds *et al.*, 1980), was uninformative because an arbitrary baseline was chosen. Hernandez-Llamas & Kimball seem to have missed the point of that table. As we originally stated, our intent was to compare the relative strengths of the cancer-site-specific associations with beer and cigarette consumption. To do so, we used the well-accepted epidemiological method of comparing cancer incidence rates (predicted by regression equations) at 2 exposure levels. The results in Table V led us to conclude that cigarette smoking was most strongly associated with cancer of the pharynx, larynx, lung (epidermoid and small-cell histological type) and bladder. This conclusion is completely consistent with the known epidemiology of cigarettes and cancer, and therefore supports the validity of the analysis. Hernandez-Llamas & Kimball are correct in stating that the percentage increases in Table V would have been different if we had used a different baseline exposure, but this point is irrelevant to our purposes, as shown in Table II herein. In this table, we have calculated the predicted percentage increase in cancer incidence due to a doubling of mean population consumption of cigarettes from 20 to 40 pack-years instead of from 10 to 20 pack-years as originally done. Again, the strongest associations are found for cancer of the pharynx, larynx, lung and bladder. When we assume a doubling of the mean population consumption of cigarettes from 5 to 10 pack-years, these same cancer sites are shown again to have the greatest percentage increase in incidence rates. Similarly, we concluded in our original paper (Hinds *et al.*, 1980) that the cancer sites most strongly influenced by mean population beer consumption were oesophagus and larynx. These conclusions are likewise compatible with past epidemiological findings on alcohol and cancer. In Table II herein, we examined the effect of doubling the mean population consumption of beer from 30 to 60 ounces per week instead of from 15 to 30 ounces, as originally done. Our original conclusions remain unaltered.

Finally, Hernandez-Llamas and Kimball stated that the characteristics of our data sets would preclude appropriate epidemological analysis. This is an assertion with implications extending far beyond our own study. Correlation studies such as ours have been published by many investigators (Stocks, 1970; Armstrong & Doll, 1975; Breslow & Enstrom, 1974; Kono & Ikeda, 1979) who used data sets which were certainly not superior to ours and in some cases, we would argue, inferior. The authors of such papers have always emphasized, as did we, that the findings of such studies should not be overinterpreted, but used primarily as indicators of hypotheses which might be tested fruitfully in more rigorous studies. Hernandez-Llamas & Kimball seem to ignore the hypothesis-generating value of such exploratory studies.

Because epidemiological studies are largely concerned with identifying causal factors of human disease, and thus deal with data from human populations, the data sets are almost always imperfect. It is not difficult to write an extensive critique of almost any epidemiological study, listing numerous shortcomings and necessary assumptions which might not be valid. However, such detailed criticism of a single epidemiological study shows a lack of understanding of the process by which causal association is established through the epidemiological method. No single epidemiological study can offer evidence strong enough to establish a causal assocation between exposure and disease. Instead, such associations become accepted only after numerous studies, conducted by different investigators in different settings and using different methods, all produce essentially the same findings. Before such multiple testing of a hypothesis can be done, however, the hypothesis must be put forward as one that warrants testing. Such hypothesis generation was exactly the purpose of our correlational study. We hope that others will not be discouraged from similar analyses which might provide leads to important environmental causes of cancer.

M. W. HINDS
J. LEE
L. KOLONEL
*Epidemiology Program,*
*Cancer Centre of Hawaii,*
*University of Hawaii, U.S.A.*
8 *April* 1982

This research was supported by Contract No. 1-N01-CP-53511 and Grant No. 1-N01-CA-15655 from the National Cancer Institute, D.H.H.S., U.S.A.

### REFERENCES

ARMITAGE, P. (1971) *Statistical Methods in Medical Research*, New York: John Wiley. p. 322.

ARMSTRONG, B. & DOLL, R. (1975) Environmental factors and cancer incidence and mortality in different countries, with special reference to dietary practices. *Int. J. Cancer*, **15**, 617.

BRESLOW, N. W. & ENSTROM, J. E. (1974) Geographic correlation between cancer mortality rates and alcohol-tobacco consumption in the United States. *J. Natl Cancer Inst.*, **53**, 631.

DOLL, R. & COOK, P. (1967) Summarizing indices for comparison of cancer incidence data. *Int. J. Cancer*, **2**, 269.

HERNANDEZ-LLAMAS, H. & KIMBALL. A. W. (1982) Critique of a study of cancer incidence and alcohol/cigarette consumption in Hawaii. *Br. J. Cancer*, **45**, 150.

HINDS, M. W., KOLONEL, L. N., LEE, J. & HIROHATA, T. (1980) Associations between cancer incidence alcohol/cigarette consumption among five ethnic groups in Hawaii. *Br. J. Cancer*, **41**, 929.

KONO, S. & IKEDA, M. (1979) Correlation between cancer mortality and alcoholic beverage in Japan. *Br. J. Cancer*, **40**, 449.

STOCKS, P. (1970) Cancer mortality in relation to national consumption of cigarettes, solid fuel, tea and coffee. *Br. J. Cancer*, **24**, 215.